# Race and Racism in Primary Care: A special collection from BJGP Open

**DOI:** 10.3399/BJGPO.2020.0175

**Published:** 2020-11-25

**Authors:** Hajira Dambha-Miller, Umar Ahmed Riaz Chaudhry, Oswald Peter Adams

**Affiliations:** 1 Editor-in-Chief, BJGP Open, London, UK; 2 Editorial Fellow, BJGP Open, London, UK; 3 Guest Editor, BJGP Open, London, UK; 4 Dean of the Faculty of Medical Sciences, University of the West Indies, Cave Hill Campus, Cave Hill, Barbados

**Keywords:** primary care, racism, race, primary healthcare, general practice, general practitioners

The *Black Lives Matter* campaign has highlighted issues of race and racism that are present in all parts of our communities and organisations. We stand in solidarity against racism in all forms and acknowledge that organisations, including those within the publishing world, need to do more. *BJGP Open* must ensure that ethnic minority voices are heard and represented within our leadership, board members, fellows, editorial staff, readers, and authors.^1^ The statistics on under-representation of ethnic groups at all levels of publication and academia make uncomfortable reading, and the reluctance to openly acknowledge this is delaying progress.^2,3^ The tokenistic phrases and well-intended platitudes on the need for greater diversity must be matched with measurable action. As a journal, we need to play our part and demonstrate a commitment to meaningful progress on this issue.

Accordingly, *BJGP Open* has set out an ambitious programme of work on race and diversity. It starts with this special issue, in which we have collated individual and collective experiences on racism in primary care alongside relevant policy and practice submissions. GPs, trainees, medical students, and academics have shared their recent lived experiences of the everyday racism that ethnic minorities face in our discipline today.^4^ These challenging experiences range from a GP being doused in alcohol by a patient;^5^ a medical student repeatedly overlooked due to race;^6^ and a GP required to ignore racial slurs at the request of a senior partner.^7^ *Where is the accountability*, they ask?

Hartland and Larkai describe in their article the concept of decolonisation in reforming the medical school curriculum, and explain why this is important in addressing unconscious biases.^8^ They highlight their approach to bringing about long-term institutional change, which they suggest is necessary to equip medical students with the tools to manage an increasingly diverse population. Bhatti and colleagues in their viewpoint provide us with another historical perspective, emphasising the substantial contribution of migrant labourers to different roles in primary care.^9^ The authors highlight the Medical Workforce Race Equality Standard (MWRES) in narrowing the gap in workplace experiences and opportunities, and call for the inclusion of general practice to bring about sustainable change. Sarfo-Annin takes this further in a commentary that outlines the case for affirmative action in reducing racial exclusion by enabling structural changes to achieve representative diversity, especially for leadership positions.^10^ They argue that the term ’BAME’ (Black, Asian and Minority Ethnic) had too broad a definition to be useful as a metric for ethnic inclusion because of significant heterogeneity in the experiences of different minority ethnic groups. While acknowledging the complex challenges, they call for more detailed data on the ethnicity of GPs to raise awareness, generate dialogue, and enhance the organisational accountability that might improve inclusivity. Ikpoh's article then presents a very personal trainee perspective.^11^ Next, Gopal and colleagues contribute a discussion on the effect of racial disparities on GPs, and question whether the non-minority workforce needs to go further in acknowledging personal prejudices and implicit biases.^12^ The authors suggest that improvement will come in the form of greater diversity in leadership and governance structures.

Their suggestions on the importance of diversity in leadership and governance have not gone unheard. At our journal, we have made strategic appointments, including a new Editorial Board which represents one of the most diverse in the history of our larger journal family. Similarly, our team of editorial fellows and medical students reflects racially diverse voices. Led by our Editorial Board, we have additionally established a new mentoring and outreach programme for students from around the world, particularly those from low sociodemographic and minority backgrounds, who are less likely to attend higher education. We offer virtual placements, student projects, and one-to-one mentoring. This is alongside a mentoring programme to support authors from low- and middle-income countries, who are less likely to have manuscripts accepted across biomedical journals. We provide opportunities for additional feedback and support in the preparation of their manuscripts.

We are additionally opening a period of consultation from 1 December 2020 to 11 January 2021 on the collection of anonymised sociodemographic data, including ethnicity, from all our authors at the point of submission. This will allow us to openly report submissions and acceptance rate by ethnicity, to identify unconscious biases, and to address these as necessary through, for example, blinding of peer reviewers and editorial staff to author details. In turn, we hope that other scientific journals might be encouraged to follow suit. We are already working with colleagues at other journals on our ‘research into publication science’ programme. We are running several studies to collate data and scrutinise evidence on publication science around sex and ethnicity. These strategic programmes are aimed at ensuring that when authors submit their work to us, it is the quality of the scientific content and not race or, indeed, sex that determines acceptance. Our efforts at the journal have been complemented by a diverse programme of work currently being carried out at the Royal College of General Practitioners (RCGP).^13^ This is summarised in an editorial included within this collection by Professor Amanda Howe (President), Professor Martin Marshall (Chair), and Dr Valerie Vaughan-Dick (Chief Operating Officer) of the RCGP. They summarise recent learning, recognise the diversity of the membership, and put forward plans on tackling racism within the discipline.

Research, alongside the lived experiences shared in this collection, emphasises that racism is very much alive within primary care, and that the world around us is shaped by structural and systemic racism. The expectation in all aspects of primary care is to maintain the highest level of professional and moral standards. It is incumbent upon all of us in primary care to be aware of the issues around race, to speak up, and to support each other in tackling this challenging issue.

If you would like to contribute to the discussion or comment on our consultation, please tweet us @BJGPOpen, respond through our online e-letters https://bjgpopen.org/letters, or email us at BJGPOpen@rcgp.org.uk.
